# Distinct topographic organization and network activity patterns of corticocollicular neurons within layer 5 auditory cortex

**DOI:** 10.3389/fncir.2023.1210057

**Published:** 2023-07-13

**Authors:** Tatjana T. X. Schmitt, Kira M. A. Andrea, Simon L. Wadle, Jan J. Hirtz

**Affiliations:** Physiology of Neuronal Networks, Department of Biology, RPTU University of Kaiserslautern-Landau, Kaiserslautern, Germany

**Keywords:** corticofugal, mouse, neocortex, two-photon imaging, primary auditory cortex, anterior auditory field, secondary auditory cortex

## Abstract

The auditory cortex (AC) modulates the activity of upstream pathways in the auditory brainstem via descending (corticofugal) projections. This feedback system plays an important role in the plasticity of the auditory system by shaping response properties of neurons in many subcortical nuclei. The majority of layer (L) 5 corticofugal neurons project to the inferior colliculus (IC). This corticocollicular (CC) pathway is involved in processing of complex sounds, auditory-related learning, and defense behavior. Partly due to their location in deep cortical layers, CC neuron population activity patterns within neuronal AC ensembles remain poorly understood. We employed two-photon imaging to record the activity of hundreds of L5 neurons in anesthetized as well as awake animals. CC neurons are broader tuned than other L5 pyramidal neurons and display weaker topographic order in core AC subfields. Network activity analyses revealed stronger clusters of CC neurons compared to non-CC neurons, which respond more reliable and integrate information over larger distances. However, results obtained from secondary auditory cortex (A2) differed considerably. Here CC neurons displayed similar or higher topography, depending on the subset of neurons analyzed. Furthermore, specifically in A2, CC activity clusters formed in response to complex sounds were spatially more restricted compared to other L5 neurons. Our findings indicate distinct network mechanism of CC neurons in analyzing sound properties with pronounced subfield differences, demonstrating that the topography of sound-evoked responses within AC is neuron-type dependent.

## 1. Introduction

The detection, recognition, and interpretation of sound is essential for orientation, communication, and survival in natural environments. Neuronal networks in the AC possess distinct response modes to categorize sensory input and extract relevant information, which reflect to at least some extent the frequency-related topography of AC neurons (Bathellier et al., 2012). Advances in two-photon imaging, providing single-cell resolution, have strongly changed the knowledge about the precision of these tonotopic gradients over the last one to two decades. Earlier works reported maps with varying degrees of local best frequency (BF) heterogeneity [Bandyopadhyay et al., 2010; Rothschild et al., 2010; Issa et al., 2014, reviewed in Kanold et al. (2014)], while recent studies, using enhanced technical and analysis tools, revealed a moderate precision of tonotopic gradients in well-tuned neurons (Romero et al., 2019; Gaucher et al., 2020). Additionally, Tischbirek et al. (2019) reported a consistent tonotopic gradient throughout all layers in primary auditory cortex (A1), while Guo et al. (2012) found a varying, though generally present tonotopy in all layers in the core AC subfields A1 and anterior auditory field (AAF) using electrical recordings. However, there is little to no information about topographical sound response properties of different neuron types within deep AC layers, partly due to optical access being technically challenging. In general, AC imaging studies have either investigated tonotopy of pyramidal neurons regardless of subclasses, or regardless of neuron type altogether. However, several studies have reported distinct physiological and morphological features of L5 neurons with regard to their projection target (Christophe et al., 2005; Hattox and Nelson, 2007; Fu et al., 2019; Takahashi et al., 2020; Rindner et al., 2022; Mohan et al., 2023; Musall et al., 2023). While regular-spiking AC neurons target cortical areas, intrinsic-bursting neurons, morphologically characterized by large cell bodies and thick tufted apical dendrites, project to subcortical targets (Sun et al., 2013; Gaucher et al., 2020; Takahashi et al., 2020). Corticofugal projections terminate in virtually all upstream central auditory stations [reviewed in Schofield (2012), Saldaña (2015), and Terreros and Delano (2015)]. They originate mainly from L5 in case of brainstem-targeting neurons, with up to 25% contribution of L6 in case of CC projections (Doucet et al., 2003; Bajo and Moore, 2005; Slater et al., 2018). Recent research described intriguing synaptic and network features of CC neurons, such as high complexity of excitatory convergence onto their dendrites, direct thalamic input, and distinct integration into intracortical circuits, suggesting that CC neurons provide quick and robust feedback signals (Kim et al., 2015; Rock and Apicella, 2015; Slater et al., 2018).

Modulatory effects of CC projections are diverse, ranging from shifting the BF of IC neurons toward the BF of activated AC neurons (Yan et al., 2005) to modulation of the sensitivity of IC neurons to sound frequency, intensity, or spatial position of the sound source (Yan and Ehret, 2002; Yan et al., 2005; Nakamoto et al., 2008; Blackwell et al., 2020). Furthermore, CC neurons are reported to play a key role in predictive coding in the IC (Malmierca et al., 2015; Lesicko et al., 2022), and to mediate learning-induced auditory plasticity (Bajo et al., 2010), and flight-response (Zingg et al., 2017; Xiong et al., 2015). This evidently high importance of CC neurons for the function of the auditory system calls for the in-depth study of their physiological properties.

Recently, Williamson and Polley (2019) reported auditory L5 corticofugal neurons to respond less selective to different sounds compared to other L5 neurons. However, topographic relation of sound-evoked responses with regard to the projection target of L5 AC neurons has not been investigated so far. In the present study, we used *in vivo* two-photon Ca^2+^ imaging employing the highly sensitive sensor jGCaMP7f (Dana et al., 2019) in mouse AC to investigate population activity patterns of CC neurons. We revealed lower topographic order of frequency representation in core AC subfields in comparison to other L5 neurons as well as widespread activity clusters with overall strong correlations between neurons and high reliability. Data obtained from A2 differed remarkably, with CC neurons displaying altogether higher topographic order than other neuron types, suggesting subfield-specific roles of CC neurons.

## 2. Materials and methods

### 2.1. Animals

All experiments were performed on C57BL/6J mice of either sex, bred in the animal facility of the University of Kaiserslautern and maintained in a 12 h light-dark cycle, with food and water available *ad libitum*. Animal experiments were approved by the Landesuntersuchungsamt of Rhineland-Palatinate under file numbers G18-2-028 and G20-2-022 according to the German Animal Protection Law (TschG § 7, Absatz 2).

### 2.2. Surgical procedures

Four to 8 weeks old animals were deeply anesthetized via isoflurane inhalation (5% initial, 1–3% during procedure). They were placed on a heating mat into a stereotactic frame. Following removal of hair, disinfection of the skin using Braunol, and systemic (5 mg/kg carprofen) and local (2% lidocaine) administration of analgesics, the skin was opened. The desired location for injection of AAV vectors was determined using stereotactic coordinates as well as landmarks (suture lines of the skull) in case of injection into the AC. A small hole was drilled into the skull using a dental drill. In case of injection into the AC, the left Musculus temporalis needed to be pushed aside or partly removed, if necessary. Vector of titers of about 1x10^13^ GC/ml was injected at 80 nl/min at the desired depth using a thin needle (NanoFil, WPI, Sarasota, FL, USA) and a micropump (UMP3, WPI). AAV vectors AAV2-retro-hSyn-jGCaMP7f, AAV1-hSyn-jGCaMP7f (Tervo et al., 2016; Dana et al., 2019), and AAV2-retro-CAG-tdTomato were ordered from Addgene (Watertown, MA, USA). pGP*-*AAV-syn-jGCaMP7f-WPRE was a gift from Douglas Kim and GENIE Project (Addgene viral prep # 104488-AAV1 and Addgene viral prep # 104488-AAVrg; RRID:Addgene_104488).^1^ pAAV-CAG-tdTomato (codon diversified) was a gift from Edward Boyden (Addgene viral prep # 59462-AAVrg; 59462; RRID:Addgene_59462).^2^ AAV1-CaMKII-jGCaMP7f was kindly provided by Dr. Christoph Körber (Heidelberg University, Germany). Five minutes after end of injection (0.25 μl for injection in IC, 0.75–1.0 μl for injection into AC, here in some cases divided into two different injections to cover a wide AC area), the needle was withdrawn, and the skin was sutured. In case of imaging experiments under awake conditions, a piece of skin was removed during the same surgical procedure and a titanium plate/anchor was attached to the skull using dental cement (C&B Metabond, Parkell Inc., Farmingdale, NW, USA), not leaving any skull exposed. Isotonic, body warm NaCl-solution was administered subcutaneously during the procedure to ensure hydration of the animal. The animals were returned to their cage and monitored daily until the final experiment. Carprofen was administered on the first 2 days following surgery (also applies to following surgical procedures). In case of dual labeling experiments, two injection procedures were performed, first injecting AAV2-retro-CAG-tdTomato into the IC, and then injecting AAV1-CamKII-jGCaMP7f into the AC 1 week later. A total of 2–3 weeks after injection of GCaMP*-*carrying vector, the animal was again anesthetized via isoflurane inhalation, analgesics were administered and general surgical procedures carried out as described above. A flap of skin covering the AC was removed. In case of imaging under anesthesia, a head plate/anchor was attached as described above. A small section of the skull was thinned using a dental drill until a small piece (about 2 mm in diameter) of skull could be removed effortlessly with fine forceps. The dura mater was removed. The exposed brain was sealed with a thin coverslip, attached with dental cement. The animal was then moved to the two-photon microscope in case of direct imaging under anesthetized conditions, or returned to the cage in case imaging experiments would commence at a later day.

### 2.3. Activity imaging and sound stimulation

Head-restrained animals were either kept under isoflurane anesthesia (0.7–1%) via a nosepiece while body temperature was maintained with a heating mat, or placed on a treadmill (Luigs and Neumann, Ratingen, Germany) for awake imaging. In case of awake imaging, the animal was habituated to the experimental setup in at least 5 short sessions of increasing length in between AAV injection and window implantation. In case of imaging under anesthetized conditions, isotonic, body warm NaCl-solution was administered subcutaneously during the experiment to ensure hydration of the animal. A two-photon microscope (Ultima Investigator, Bruker AXS SAS, France) with a titling objective mount was used, allowing upright position of the animals’ head while recording activity in the left AC. The beam of a Ti:Sapphire fs-pulsed laser (Chameleon Vision II, Coherent Europe, The Netherlands) was focused through a 20 × 1.0 NA (XLUMPLFLN20XW, Olympus Europe, Germany) or 16 × 0.80 NA objective (CFI75 LWD, Nikon GmbH, Düsseldorf, Germany), using 940 nm excitation for GCaMP*-*based activity imaging and 1,040 nm excitation for identification of tdTomato-labeled neurons. Activity imaging was performed at about 30 fps using a resonant scanner. Emitted light was collected with GaAsP photomultiplier tubes using appropriate emission filters. Imaging was controlled by Prairie View v5.5 (Bruker). The imaging depth ranged from 520 to 800 μm below the pial surface. Widefield illumination was provided by a blue 470 nm LED (Thorlabs GmbH, Bergkirchen, Germany). Here emitted light was collected by a 10 × 0.30 NA (UMPLFLN, Olympus) or 4 × 0.20 NA (Thorlabs) water-immersion objective using an appropriate green emission filter and recorded with a scientific CMOS camera (Prime 95B, Teledyne Photometrics, Tucson, AZ, USA), controlled by Micro-Manager v.2.0. Sampling rate was 10 Hz and the resulting field of view (FOV) size comprised 2,048 pixels x 2,048 pixels. All widefield FOVs combined covered ∼1.5 mm x 1.5 mm. Imaging plane was set to ∼200 μm below the pial surface. In all experiments, the objective was rotated by 45° while the animal was maintained in an upright head-fixed position. Imaging was performed inside a light-tight sound attenuation chamber (ambient noise level (above 2 kHz) < 20 dB, resonant scanner noise at 8 kHz < 30 dB). Stimuli were generated with a 24-bit digital-to-analog converter (RX6 Multifunction Processor, Tucker-Davis Technologies (TDT), Alachua, FL, USA) using scripts programmed in RPvdsEX v88 (TDT) controlled by Matlab 2019a (MathWorks, Natick, MA, USA). Stimuli were presented via a free-field speaker (ES1, TDT), positioned ∼10 cm from the right (contralateral) ear of the mouse and powered by an electrostatic speaker driver (ED1, TDT). Free-field stimuli were calibrated before each recording session using a microphone (Model 378C01, PCB Piezotronics, Depew, NY, USA) amplified by a MA3 (TDT) and controlled by SigCalRP v4.2 (TDT). Based on this calibration, sound pressure levels (SPLs) were adjusted by applying an online FIR-filter. Sound stimulation was triggered by the first acquired frame (start of scanning in case of two-photon imaging, start of first camera frame in case of widefield imaging). During two–photon imaging, 17 different pure tones (PTs) were presented randomized (4–64 kHz, 4 equivalent steps per octave) in 10 repetitions of increasing intensities (30–70 dB SPL in 10 dB steps, new recording started for each level). Tone duration was 250 ms followed by a 1 s pause. For complex acoustic stimulation 10 different animal sounds (Blue tit, Tree creeper, Vervain hummingbird, Blasius’s horseshoe bat, Common noctule bat, Meadow grasshopper, Mottled grasshopper, Great green bush-cricket, Lesser field grasshopper, European water shrew)^3^ were presented pseudorandomized, 10 times each with a pause of 1 s between vocalizations. Because these stimulations varied in length, we limited the analysis window to the first 550 ms of each sound. The maximal duration of a single recording was 5 min 13 s. We thank Matthias Göttsche (Stocksee, Germany) for allowing us to use the recordings of the Blasius’s Horseshoe Bat. For widefield recordings 5 different frequencies (4–64 kHz, 1 octave steps) were presented pseudorandomized at 70 dB for 500 ms followed by a 5 s pause, 16 times each.

### 2.4. Transcardial perfusion and preparation of histological brain slices

Animals not undergoing the procedure described in the following paragraph were sacrificed after the final imaging experiment via decapitation under deep anesthesia (3–5% isoflurane).

A total of 2–4 weeks after injection of viral vectors, some mice were intraperitoneally injected either with a lethal dose of 7% chloral hydrate (700 mg/kg body weight) or ketamine/xylazine (220/24 mg/kg body weight). As soon as the mice were pain and reflex free, the thorax was opened. A winged infusion needle (0.05 mm) connected to a peristaltic pump (Ecoline VC-360, Ismatec, Cole-Parmer GmbH, Wertheim, Germany) was inserted into the left ventricle to perfuse the circulatory system with phosphate-buffered saline (PBS) containing (in mM) 130 NaCl, 7 Na_2_HPO_4_ (2 H_2_0) and 3 NaH_2_PO_4_ (H_2_0) (pH adjusted to 7.4 with NaOH). Immediately after starting the peristaltic pump, a small incision was made into the right atrium to allow the outflow of blood and PBS. When the blood was completely removed, 4°C-cold 4% paraformaldehyde in 0.1 M phosphate buffer (NaH_2_PO_4_ and Na_2_HPO_4_, 1:1, pH 7.4) was pumped through the vascular system for 20–30 min. Next, the brain was removed from the skull and post-fixed in paraformaldehyde at room temperature for 2 h and incubated overnight at 4°C in 30% sucrose in PBS.

For slice preparation, a coronal cut was performed at bregma using a razor blade. The brain block was then positioned with the cut surface on the sample holder of a sledge microtome (MICROM HM 430, Thermo Fisher Scientific GmbH, MA, USA) and frozen in 30% sucrose in PBS at ∼−18°C. 100 μm thick coronal slices containing the brainstem, cerebellum and cortex were cut and collected in 15% sucrose in PBS and washed three times in PBS for 10 min. Finally, the slices were mounted on gelatin-chromium potassium sulfate-coated microscope slides, embedded in self-made medium containing 2.5% 1,4-diazabicyclo[2.2.2]octane.

Injection sites and expression patterns were documented using an Axio-Scope 2 (Carl Zeiss AG, Oberkochen, Germany), with 2.5-fold dry (Fluar, 0.12 NA) and 10-fold dry (Plan-Neofluar, 0.3 NA) objectives. Images were visualized and captured with a scientific CMOS camera (Kiralux, Thorlabs GmbH) controlled by the software package Micro-Manager v1.4 (freely available).^4^ Fluorophores were excited with either blue light (450–490 nm) or green light (540–552 nm) using appropriate emission filters. Contrast of images was enhanced within Micro-Manager.

### 2.5. Widefield analysis

The procedure of image processing was adapted from Romero et al. (2019). Raw images were downsampled to a 256 pixel x 256 pixel resolution. Motion correction was carried out using NoRMCorre (Pnevmatikakis and Giovannucci, 2017). Small drifts in fluorescence signal were removed by computing a temporal baseline (F_0_) for each pixel from a polynomial fit (degree 3) of a 15 s sliding window (Chronux toolbox, Matlab). The change in fluorescence was calculated for each frame as percent change from the temporally smoothed signal ($\frac{△\,\text{F}}{{\text{F}}_{0}}\cdot 100)$. These amplitudes were used for further analysis. Baseline activity levels for each stimulus were defined for each pixel by creating a histogram of amplitudes of all frames during the 2 s prestimulus period. To check for tone-evoked responses, the maximum amplitude was picked from the 750 ms post-stimulus onset period and averaged with the preceding and following frame. In cases where the resulting value exceeded the prestimulus baseline activity distribution by at least 2 standard deviations (z-score > 2), the response was characterized as tone–evoked. A frequency specific response amplitude was only calculated when a tone-evoked response occurred in a minimum number of repetitions, dependent on the experimental approach. In experiments with CC-limited GCaMP7f expression, a tone-evoked response had to occur in at least 2 out of 16 repetitions. In experiments with general GCaMP7f expression, the minimum number of tone–evoked responses to a certain frequency was 4 of 16 repetitions. The frequency-specific response was then calculated as the mean of all significant tone-evoked response amplitudes to the respective frequency. In the end, all frequency-specific responses were compared. The frequency eliciting the highest mean response amplitude within a pixel was set as the BF of that given pixel. Custom-written code for these all well as other analyses carried out in this study can be found online at github.com/HirtzLab/Imaging_auditory_corticofugal_L5.

As one FOV acquired with the 10x objective covered only a part of the AC, multiple overlapping FOVs were necessary in order to create a gapless BF map. The frequency-specific response amplitudes of each pixel within a FOV were normalized to provide comparability. In cases where one pixel was represented more than once (due to overlapping FOVs), the BF with the higher normalized mean response amplitude was chosen.

Next, a vector-based calculation of reversal points, similar to the analysis in Romero et al. (2019), was provided as follows to assist subfield parcellation. First, centers of existing low-frequency hubs were identified. From each of these a set of 1,440 radial vectors from 0 to 360° (0.25° step size) were drawn. The mean BFs along each radial vector (±1°) were smoothed with a moving average (window size 10 frames). The smoothed values were then fitted with a gaussian filter (degree 3), so that reversal points (first maxima) could be marked in the BF map. The end of the AC was defined as the point, where 10 pixels in a row showed no sound-evoked response at all. The marked reversal and end points within the BF map served as a template for the “drawassist” function of Matlab. Thereby, the subfield borders could be drawn by hand, but the outline was corrected by the information of the underlying BF map. Assignment of A1, AAF, and A2 was performed, based on existing knowledge from earlier studies (Tsukano et al., 2015, 2016; Romero et al., 2019).

In experiments under anesthetized conditions where GCaMP7f expression was limited to CC neurons, a less clear BF map was observed. To facilitate subfield parcellation in these cases, a template widefield map was created. First, borders and areas of A1, AAF, and A2 were determined from 4 animals expressing GCaMP7f, either in pyramidal neurons or all neurons across multiple AC layers, displaying clear BF maps. These were aligned at their center points (cross point between the three subfields). A border template was drawn for each subfield, with 50% of the respective subfield area present. If needed, this template was then projected on the calculated BF map in CC-limited experiment to allow a more assured drawing of subfield borders.

### 2.6. Tuning and topography analysis

All two-photon imaging data were processed with the publicly available software package Suite2p (Pachitariu et al., 2017; Stringer and Pachitariu, 2019)^5^ with optimized processing parameters. Movement correction, cell detection, neuronal and neuropil trace extraction, and spike deconvolution was performed automatically. In brief, images were registered to correct for brain movement by phase-correlation. A region of interest (ROI) was detected by an iterative algorithm finding clustered, correlated pixels that fit a model of ROI activity. A ROI’s uncorrected fluorescence trace was extracted as the sum of the somatic signal, neuropil signal (scaled by a ROI-specific coefficient), and noise contribution. The neuropil contribution is estimated by averaging signal within an annular ring surrounding the ROI and subtracted from the uncorrected fluorescence trace. Spike deconvolution was performed on the neuropil-corrected fluorescence trace based on the OASIS algorithm (Friedrich et al., 2017). Finally, ROIs were identified as “cell” or “non-cell” based on a quality control dependent on activity statistics (skewness, variance, correlation to surrounding pixels) and anatomical shape (area, aspect ratio).

To define ROIs as “responsive,” mean spiking probabilities during the 400 ms pre- and post-stimulus onset periods were calculated. To check for a significant response, a one-way ANOVA comparing pre- and post-stimulus onset spiking probability in all 85 frequency-intensity combinations was performed. If *p* > 0.01 in every combination, the ROI would be defined as “non-responsive” and excluded from further analysis. The preselection was performed automatically by a custom-made Matlab script.

Next, the mean post-stimulus onset spiking probability in response to the different PTs at each SPL was calculated. The means over all spiking probability levels at the different SPLs were calculated for each presented frequency. Subsequently, these data points were fitted with a unimodal gaussian (Equation 1 | gauss1) and bimodal gaussian (Equation 2 | gauss2) fit function to check for the existence of a single- or double-peak tuning, respectively.


(1) $$g\,a\,u\,s\,s\,1=A_{1}\cdot e^{-{\left(\frac{x-B_{1}}{C_{1}}\right)}^{2}}+D$$

(2) $$g\,a\,u\,s\,s\,2=A_{1}\cdot e^{-{\left(\frac{x-B_{1}}{C_{1}}\right)}^{2}}+A_{2}\cdot e^{-{\left(\frac{x-B_{2}}{C_{2}}\right)}^{2}}+D$$

Because of different numbers of parameters of the two fits, the adjusted coefficient of determination (*R*^2^_*adj*_) was used to determine the best match. If both fits resulted in a *R*^2^_*adj*_ < 0.3, the neuron was classified as “irregular.” The fit resulting in a higher *R*^2^_*adj*_ determined the characterization of neuron as single- or double-peaked, respectively (see Figure 2B). The tuning bandwidth (BW) was defined as the full width at half maximum (Equation 3 | BW) of the respective tuning peak.


(3) $$\,\text{BW}=2\cdot \sqrt{2\cdot \log\left(2\right)\cdot \frac{{\text{C}}_{1/2}}{\sqrt{2}}}$$

A neurons’ BF was defined as the tone frequency which elicited the strongest significant response regardless of SPL (Hackett et al., 2011; Bowen et al., 2020). All responsive neurons were then aligned to the widefield BF map, which enabled determination of the neurons’ locations in a subfield. Global coordinates from each neuron were used to calculate the local BF distribution. For each neuron, its BF and the BFs of all neurons within 100 μm were extracted. Then, the interquartile range (IQR) of the distribution was calculated as a measure of local heterogeneity. If less than 5 neurons were within 100 μm radius (including the center neuron), no IQR was calculated. Center frequency (CeF) was defined as the peak of the single unimodal gaussian fit. To reduce the CC neuron dataset (Supplementary Figure 3), 50–80% of neurons per subfield observed were randomly excluded before calculating IQR.

### 2.7. Activity correlation analysis

To identify cells with similar activity patterns upon complex sound stimulation, a correlation analysis was performed similar to the study of Bathellier et al. (2012). Each FOV was analyzed with a custom-made Matlab script as follows. First, the average deconvolved spiking probability of a neuron was calculated. This window was 400 ms for PTs (matching the time window for tuning analysis) and 550 ms for animal vocalizations. These were lined up and transformed into a “sound vector” for each repetition. These vectors were then correlated across repetitions (Pearson correlation), both for each given neuron to determine reliability of responses as well as between the different neurons to determine their response similarity. The mean correlation value across repetitions was then used for hierarchical clustering of the cells within one FOV. Clusters were defined from a hierarchical cluster tree using dynamic tree cut (Langfelder et al., 2008; method “hybrid,” deepsplit set to 0.5) in RStudio. For shuffling, the activity of single neurons to specific sounds was assigned randomly before each correlation calculation. This process was repeated five times for each FOV. Clusters in original data were excluded if their mean correlation did not exceed the averaged correlations of all clusters resulting from shuffling by at least 2 standard deviations. This way, clusters formed as a result of random correlations at noise level were disregarded for further analysis.

### 2.8. Data visualization and statistics

Bar graphs and text present mean ± standard error of mean with number in bar depicting n-number, box plots show the mean (small rectangle), median (horizontal line), interquartile range (box), and standard deviation (whisker range). Statistical analysis was performed with Origin 2019 or Matlab 2020a–2022a. Normal distribution was tested with Kolmogorov-Smirnov test. Normally distributed data sets were compared using unpaired, two-tailed *t*-tests. Distribution-free data sets were compared using Mann-Whitney U–tests. Significance levels are as follows: *p* < 0.05 *, *p* < 0.01 **, *p* < 0.001 ***. Bonferroni *post hoc* correction of *p*-values was carried out in case of multiple comparisons.

## 3. Results

### 3.1. CC neurons display common tuning properties

**FIGURE 1:**
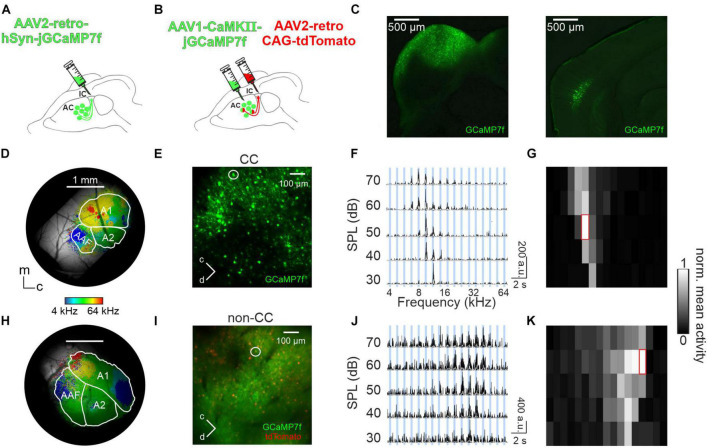
Imaging PT responses in AC L5. **(A)** Schematic depicting injection of AAV2-retro-hSyn-jGCaMP7f into the IC to image activity in auditory CC neurons. **(B)** Schematic depicting injection of AAV2-retro-CAG-tdTomato into the IC to label auditory CC neurons and additional injection of AAV1-CaMKII-jGCaMP7f into the AC to image activity from pyramidal AC neurons. **(C)** Left: GCaMP7f expression (green) at injection site in a histological brain slice containing the IC. Right: Histological brain slice of the same animal containing retrogradely labeled IC-projecting GCaMP-expressing neurons in the ipsilateral AC. **(D)** Example of a cranial window with GCaMP7f-expression in CC neurons in an awake animal [experimental approach shown in panel **(A)**], superimposed with BF for each pixel and borders of A1, AAF, and A2. m = medial, c = caudal. **(E)** Example cortical field of CC neurons imaged 533 μm below the pial surface. **(F)** Deconvolved calcium traces of 10 repetitions (black) and mean (white) of exemplary CC neuron shown in panel **(E)** (white circle) in all frequency-SPL combinations. PT duration is depicted by blue bars. **(G)** FRA showing mean activity during 400 ms after each stimulus onset, red frame indicates BF. **(H)** Like **(D)**, but example of an anesthetized animal with GCaMP7f expression in all pyramidal neurons [double-labeling approach shown in panel **(B)**]. **(I)** Like **(E)** but showing an exemplary non-CC neuron (white circle) recorded 630 μm below the pial surface. **(J,K)** Like **(F,G)**, but for non-CC neuron shown in panel **(I)**.

To study topographical activity patterns of CC neurons, the largest subset of auditory corticofugal neurons, AAV2-retro-GCaMP7f was injected into the IC of young adult mice (Figures 1A, C). A total of 2–3 weeks later, a cranial window was implanted over the ipsilateral AC. GCaMP expression was limited to L5 neurons, with no observable cell bodies in L6 (Figure 1C, right). Two-photon and widefield Ca^2+^ imaging was performed in order to characterize sound response patterns of L5 CC neurons and assign them to corresponding AC subfields, respectively. Widefield imaging in 3 anesthetized (light isoflurane) as well as 3 awake mice revealed commonly observed high and low frequency hubs (e.g., Tsukano et al., 2016; Romero et al., 2019; Tischbirek et al., 2019), demonstrating the rough-scale tonotopy of CC neurons, thus allowing for identification of AC subfields (A1, AAF, and A2; see Figures 1D, H). It should be noted that subfield identification in anesthetized animals was in some cases challenging and required us to employ a strategy of aligning the obtained maps to a “master map,” acquired from animals expressing GCaMP7f in AC neurons under hSyn or CamKII promoter, irrespectively of subtype and layer (see “2. Materials and methods” section for details). Next, we tested for tuning and tonotopy of CC neurons on single cell level, employing two-photon imaging. An exemplary FOV is shown in Figure 1E. Using 17 PTs at 5 SPLs, we recorded Ca^2+^ traces, subsequently deconvolved spike traces, and calculated the frequency response areas (FRA) of PT-responsive CC neurons (Figures 1F, G and Supplementary Figure 1). In additional sets of experiments in 4 anesthetized and 3 awake animals, we were able to distinguish between CC and surrounding (non-CC) pyramidal neurons and investigate their tuning properties separately. Labeling of CC neurons was achieved by injecting AAV2-retro-tdTomato into the IC. GCaMP7f was expressed under the promoter of CaMKII via injection of AAV1 directly into the AC, thus allowing for activity imaging of pyramidal neurons in the green channel, and identification of CC neurons in the red channel (“double labeling approach,” Figures 1B, I). This way, activity could be recorded from both neuron types separately (Figures 1J, K). Furthermore, the presence of red labeled neurons ensured that we limited our analysis to L5. While a certain amount of false negative labeling of CC neurons (and thus false positive identification of non-CC neurons as well) cannot be excluded, we found that CC neurons were identified at a rate of 49% (13 FOVs analyzed in 3 animals recoded under awake conditions, Supplementary Figure 2), which is in line with 47% reported by Sun et al. (2013), demonstrating that this error was fairly minimal. This also holds true when limiting the dataset to PT-responsive neurons (see “2. Materials and methods” for details), determining an occurrence of 43% CC neurons. When combining both labeling approaches, 56% of non-CC neurons imaged (1,623 out of 2,850) and 69% (2,399/3,464) of CC neurons were found to be PT-responsive in anesthetized animals. Similarly, under awake conditions 40% (303/755) of non-CC and 58% (2,052/3,552) of CC neurons were PT-responsive.

**FIGURE 2:**
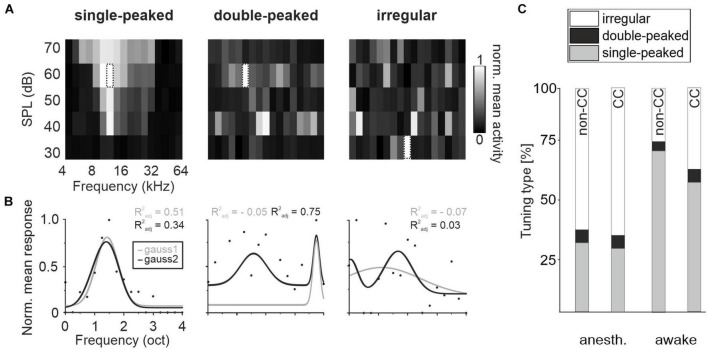
Tuning variety of AC L5. **(A)** Exemplary FRAs for a single-peaked, double-peaked and irregular tuned neuron under awake conditions, showing the mean activity of all repetitions for 400 ms after each stimulus onset. Dotted frame indicates BF. **(B)** Fit of mean responses for the three exemplary neurons over all SPLs at each stimulus frequency with bimodal gaussian (dark) and unimodal gaussian (light) to determine tuning type. Dots depict normalized mean response at BF. **(C)** Amount of single-peaked, double-peaked, or irregular tuned CC and non-CC neurons in anesthetized and awake animals.

**FIGURE 3:**
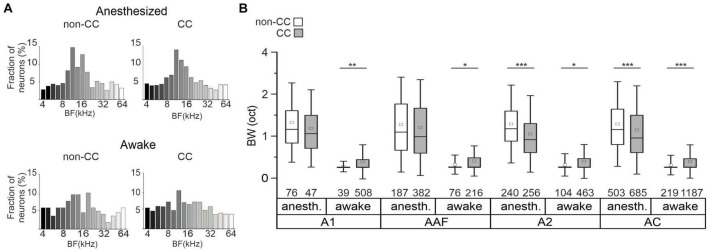
BF distribution and tuning in AC L5. **(A)** Distribution of neurons’ BF under anesthetized and awake conditions. **(B)** Box plots showing the tuning BWs in single-peaked non-CC and CC neurons under awake and anesthetized conditions in each subfield and in pooled datasets independent of subfields. Number of neurons is depicted below each box. Significance levels for comparison of anesthetized to awake conditions are not depicted, see text for details.

Auditory cortex neurons in L2/3 can basically be characterized into three tuning types: “Single-peaked neurons” with one clear response peak, “double-peaked neurons” with two response peaks at distinct frequencies or “irregular neurons” with complex FRAs, containing either three response peaks or no definable pattern at all (Gaucher et al., 2020). We also detected these tuning types in L5 (Figures 2A, B). In anesthetized animals, a moderate amount of L5 CC neurons was PT-tuned, 28.5% single-peaked and 5.9% double-peaked, with a considerably higher number in awake animals, 57.8 and 5.7%, respectively. Non-CC neurons displayed overall a slightly higher number of tuned neurons (30.9% single-peaked, 6% double-peaked in anesthetized, and 72.3 and 4% in awake mice). The remaining fractions displayed no clear tuning (Figure 2C). The BF distributions of single-peaked neurons was quite even across all frequencies tested (Figure 3A).

To further characterize FRAs with respect to different AC subfields, we calculated tuning widths of PT-responsive single-peak neurons in A1, AAF, and A2. In awake animals, tuning bandwidth was higher for CC neurons in all subfields, as well as in the pooled dataset (A1: non-CC: 0.27 ± 0.01, *n* = 39; CC: 0.38 ± 0.01, *n* = 508; *p*-value = 0.006; AAF: non-CC: 0.32 ± 0.02, *n* = 76; CC: 0.41 ± 0.02, *n* = 216; *p*-value = 0.01; A2: non-CC: 0.31 ± 0.02, *n* = 104; CC: 0.40 ± 0.01, *n* = 463; *p*-value = 0.01; pooled: non-CC: 0.31 ± 0.01, *n* = 219; CC: 0.40 ± 0.01, *n* = 1,187; *p*-value = 2.54e-5; Figure 3B). In contrast, data obtained in anesthetized animals showed a lower bandwidth of CC neurons in A2 and the pooled datasets (A1: non-CC: 1.32 ± 0.07, *n* = 76; CC: 1.18 ± 0.09, *n* = 47; *p*-value = 0.36; AAF: non-CC: 1.27 ± 0.05, *n* = 187; CC: 1.2 ± 0.04, *n* = 382; *p*-value = 0.39; A2: non-CC: 1.28 ± 0.04, *n* = 240; CC: 1.05 ± 0.04, *n* = 256; *p*-value = 5.94e-7; pooled: non-CC: 1.28 ± 0.03, *n* = 503; CC: 1.14 ± 0.03, *n* = 685; *p*-value = 7.8e-9, Figure 3B). In general, datasets obtained in anesthetized animals showed much broader tuning with high variability compared to awake condition, both for non-CC neurons (A1: *p*-value = 1.12e-17; AAF: *p*-value = 5.02e-29; A2: *p*-value = 6.33e-44; pooled: *p*-value = 1.03e-88) and CC neurons (A1: *p*-value = 1.87e-23; AAF: *p*-value = 2.86e-79; A2: *p*-value = 2.75e-84; pooled: *p*-value = 7.26e-222). These results, in addition to a generally lower number of PT-tuned neurons, led us to the conclusion that under our experimental conditions, anesthesia influenced L5 AC activity considerably. We thus limited further analysis to data obtained in awake animals to ensure our conclusions reflect AC processing under physiologically relevant conditions.

### 3.2. Weaker topographic organization of CC neurons compared to non-CC neurons in core AC

**FIGURE 4:**
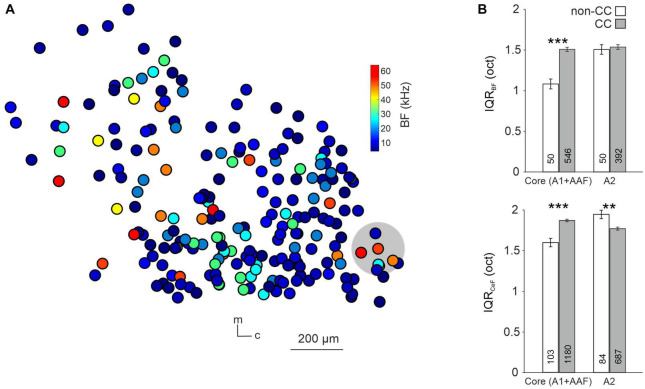
Local tuning heterogeneity of L5 non-CC and CC neurons. **(A)** Map of CC neurons within A1. Color code depicts the BF of neurons, gray circle depicts area used to calculate interquartile range of one randomly chosen neuron. m = medial, c = caudal. **(B)** Quantification of IQR_BF_ (top) and IQR_CeF_ (bottom) within core subfields and A2.

It has been shown that tonotopy is consistent throughout A1 layers (Tischbirek et al., 2019), yet it has not been investigated in other subfields of L5, nor regarding different L5 neuron types. To do so, we analyzed local tuning heterogeneity, an approach used before to indirectly quantify AC tonotopy (Bowen et al., 2020; Liu and Kanold, 2021). For each given neuron we calculated the IQR of the tuning of neurons within a 100-μm radius, focusing first on the BF of well-tuned (single-peaked) neurons in awake animals (Figure 4A). Due to us imaging across wide areas of the AC, and this analysis requiring a certain number of neurons per ring (see “2. Materials and methods” for details) we pooled data from the core AC regions A1 and AAF. IQR was higher for CC compared to non-CC neurons in core subfields, yet not in A2 (Core: non-CC: 1.08 ± 0.06 oct, *n* = 50; CC: 1.51 ± 0.03 oct, *n* = 546; *p*-value = 1.1e-04; A2: non-CC: 1.50 ± 0.06 oct; *n* = 50; CC: 1.53 ± 0.03 oct, *n* = 392; *p*-value = 0.92, Figure 4B). To investigate this aspect further, we extended our analysis to all PT-responsive neurons, determining the CeF of each neuron. This parameter calculates the frequency at the peak of a single Gaussian fit and thus provides a measure for each neuron’s overall preferred frequency, as, unlike BF, it takes the complete FRA into account. Local heterogeneity based on CeF was higher for CC neurons in core AC as well, yet actually lower in A2 (Core: non-CC: 1.60 ± 0.05 oct, *n* = 103; CC: 1.87 ± 0.02 oct, *n* = 1,180; *p*-value = 7.9e-06; A2: non-CC: 1.95 ± 0.05 oct, *n* = 84; CC: 1.77 ± 0.02 oct, *n* = 687; *p*-value = 0.0092). A possible caveat of this analysis is that we recorded a higher number of CC neurons than non-CC neurons, which could bias the results. We thus randomly excluded neurons from the CC dataset (before performing the calculation of local heterogeneity) until sample sizes were more comparable. Resulting IQR values differed very little from our initial dataset, both for BF (Core: non-CC: 1.08 ± 0.06 oct, *n* = 50; CC: 1.53 ± 0.05 oct, *n* = 75; *p*-value = 2.6e-04; A2: non-CC: 1.50 ± 0.06 oct; *n* = 50; CC: 1.41 ± 0.07 oct, *n* = 65; *p*-value = 0.49, Supplementary Figure 3A) and CeF (Core: non-CC: 1.60 ± 0.05 oct, *n* = 103; CC: 2.00 ± 0.03 oct, *n* = 228; *p*-value = 2.2e-08; A2: non-CC: 1.95 ± 0.05 oct; *n* = 84; CC: 1.57 ± 0.06 oct, *n* = 96; *p*-value = 4.2e-04, Supplementary Figure 3B). Overall, these results indicate lower tonotopy in core AC subfields for CC neurons compared to non-CC neurons, yet not within A2.

### 3.3. CC neurons form strong and widespread clusters during sound processing

**FIGURE 5:**
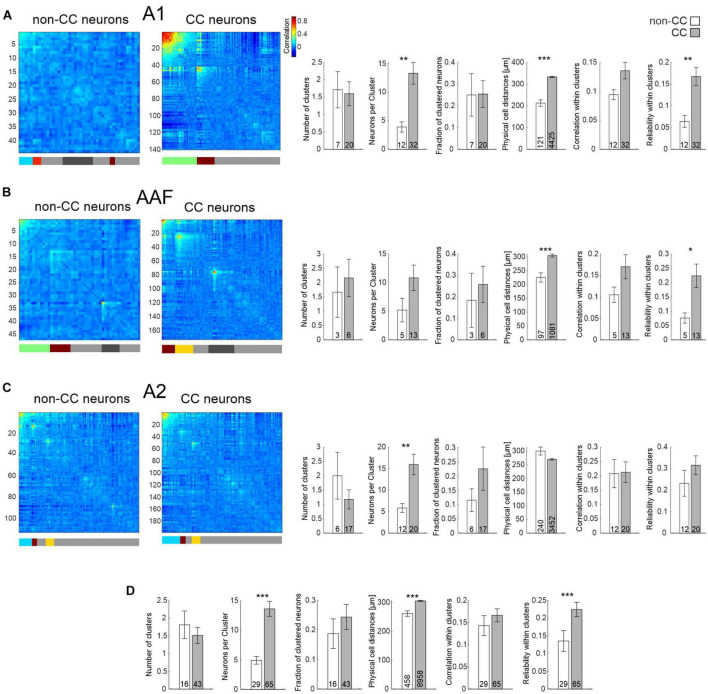
Network analysis of non-CC and CC neurons in response to PTs. **(A)** Left: Hierarchical clustering of cell correlations of exemplary FOVs in A1 in response to 17 PTs. Diagonal line depicts reliability of neurons. Colors in the bar below depict clusters. Neurons not belonging to any cluster are depicted as gray in the bar below. Light gray depicts those not included into clusters by the initial clustering, dark gray depicts clusters which have been excluded due to low correlation values. Correlation color scale shown on the right is also applicable for **(B,C)**. Right: Statistics comparing network activity features of non-CC and CC neurons within A1. N-number are FOVs, clusters, or number of all neuron pairs within clusters in case of physical cell distance. **(B)** As A, but for AAF. **(C)** As A, but for A2. **(D)** Statistics comparing network activity features of non-CC and CC neurons after pooling data from all subfields.

We next analyzed population activity patterns of non-CC and CC neurons in awake animals. Neuronal responses of single neurons to 17 PTs (same frequencies as described above) at 50 dB were averaged across an analysis window of 400 ms. Similar to the approach used by Bathellier et al. (2012), the responses to the different sounds were concatenated for each neuron for each repetition, allowing to correlate the activity between cell pairs, and also providing a measure for reliability of responses (diagonal of correlation matrices shown in e.g., Figure 5A).

When analyzing sound clustering of CC and non-CC neurons, including data from animals expressing GCaMP7f only in CC neurons, we found that CC neurons overall formed activity clusters which consisted of more neurons compared to non-CC neuron clusters, although the difference was not significant for AAF (Figures 5A–C and Table 1). The fraction of clustered neurons within a FOV and the number of clusters were not different. Interestingly, the physical distance between neuron pairs belonging to a given activity cluster was higher for CC neurons compared to non-CC neurons within A1 and AAF. These observations are in line with our results regarding local heterogeneity, demonstrating a less topographically organized population activity of CC neurons in core AC. This difference was not found for data obtained in A2. In fact, a slight tendency toward lower distances for CC neurons was observed in line with similar or lower local heterogeneity between the two neuron types, depending on the dataset analyzed. Correlation values between different neurons within clusters did not differ significantly between the neuron types yet displayed a strong tendency to be higher for CC neurons in A1 and AAF. The reliability of neuronal activity was higher in A1 and AAF and when pooling datasets across subfields (Figure 5D). Detailed information about all statistical results is given in Table 1. These results are in agreement with CC neurons showing a broader tuning, and thus forming larger clusters than non-CC neurons, and speak for reliable network activity of CC neurons. It should be noted that in general the dataset contained more CC than non-CC neurons. However, the average number of neurons per FOV did not differ between the two neuron types (non-CC: 57 ± 12; *n* = 16; CC neurons: 78 ± 10; *n* = 43; *p*-value = 0.63, FOVs divided into multiple in case of being composed of more than one subfield), dissipating the concern that some of the differences observed might be due to differences in neuron number.

**TABLE 1:** Quantification of CC and non-CC neuron clusters in L5 in response to 50 dB PTs.

	A1		AAF		A2		All subfields	
	**non-CC**	**CC**	**non-CC**	**CC**	**non-CC**	**CC**	**non-CC**	**CC**
No. of clusters	1.7 ± 0.5 (7)	1.6 ± 0.3 (20)	1.7 ± 0.9 (3)	2.2 ± 0.7 (6)	2 ± 0.8 (6)	1.2 ± 0.3 (17)	1.8 ± 0.4 (16)	1.5 ± 0.2 (43)
*p*-value	0.8636		0.6682		0.3271		0.4977	
No. of neurons per cluster	3.9 ± 0.9 (12)	13.3 ± 1.9 (32)	5.2 ± 2.1 (5)	10.8 ± 2.2 (13)	5.8 ± 1 (12)	16 ± 2.4 (20)	4.9 ± 0.7 (29)	13.6 ± 1.3 (65)
*p*-value	0.0051		0.1624		0.0034		3.5e-06	
Fraction of clustered cells	0.25 ± 0.1 (7)	0.25 ± 0.06 (20)	0.18 ± 0.13 (3)	0.26 ± 0.08 (6)	0.12 ± 0.04 (6)	0.23 ± 0.08 (17)	0.19 ± 0.05 (16)	0.24 ± 0.04 (43)
*p*-value	0.9739		0.6331		0.4136		0.4631	
Phys. distance (μm)	213 ± 15 (121)	333 ± 3 (4,425)	226 ± 17 (97)	305 ± 6 (1,081)	301 ± 15 (240)	270 ± 3 (3,452)	262 ± 10 (458)	305 ± 2 (8,958)
*p*-value	3.9e-14		4.5e-06		0.97		3.8e-12	
Correlation	0.1 ± 0.01 (12)	0.14 ± 0.01 (32)	0.11 ± 0.02 (5)	0.17 ± 0.03 (13)	0.21 ± 0.05 (12)	0.21 ± 0.04 (20)	0.14 ± 0.02 (29)	0.17 ± 0.02 (65)
*p*-value	0.1169		0.1872		0.9376		0.1384	
Reliability	0.06 ± 0.01 (12)	0.17 ± 0.02 (32)	0.08 ± 0.02 (5)	0.22 ± 0.04 (13)	0.23 ± 0.06 (12)	0.32 ± 0.04 (20)	0.14 ± 0.03 (29)	0.22 ± 0.02 (65)
*p*-value	0.0054		0.0460		0.2598		7e-04	

Data obtained from 6 awake animals. Values are shown as mean ± S.E.M. n-number is depicted in parentheses.

**FIGURE 6:**
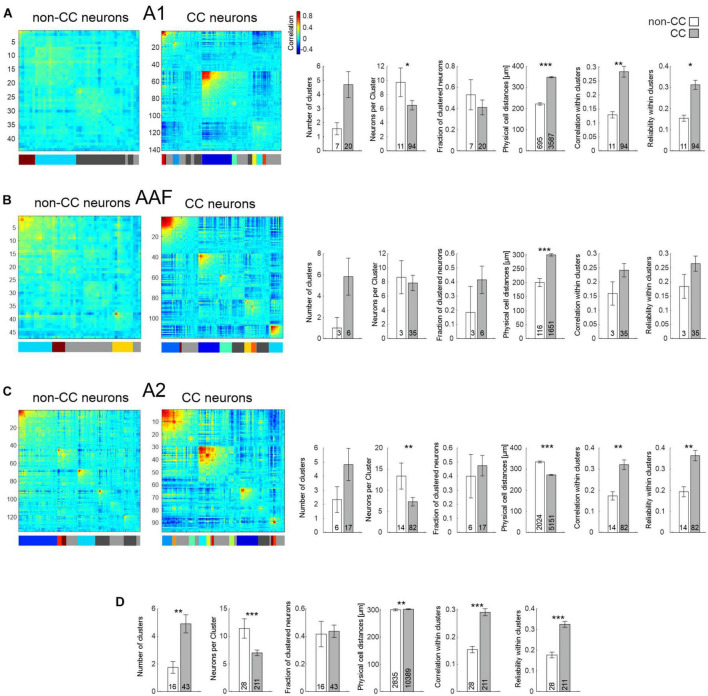
Network analysis of non-CC and CC neurons in response to complex sounds. **(A)** Left: Hierarchical clustering of cell correlations of exemplary FOVs in A1 in response to 10 animal vocalizations. Diagonal line depicts reliability of neurons. Colors in the bar below depict clusters. Neurons not belonging to any cluster are depicted as gray in the bar below. Light gray depicts those not included into clusters by the initial clustering, dark gray depicts clusters which have been excluded due to low correlation values. Correlation color scale shown on the right is also applicable for **(B,C**). Right: Statistics comparing network activity features of non-CC and CC neurons within A1. N-number are FOVs, clusters, or number of all neuron pairs within clusters in case of physical cell distance. **(B)** As A, but for AAF. **(C)** As A, but for A2. **(D)** Statistics comparing network activity features of non-CC and CC neurons after pooling data from all subfields.

While using PTs to characterize AC network patterns is valuable in the context of tuning and tonotopy it nevertheless is based on a rather artificial paradigm. Thus, in a next step, we used complex acoustic stimulations consisting of 10 animal vocalizations with rich spectral and harmonic content. Results obtained from the same FOVs were similar to those obtained using PT stimulation in such as the physical distance of neurons within clusters was higher for CC neurons in A1 and AAF (Figures 6A, B and Table 2), yet even significantly lower in A2 (Figure 6C). Furthermore, correlations and reliabilities in clusters were higher for CC neurons within A1 and A2, and when pooling data across all subfields (Figure 6D). However, in contrast to data obtained using PTs, CC neurons overall appeared to form more clusters containing less neurons compared to non-CC neuron clusters, though most of these observations were only statistically significant when pooling data across subfields.

**TABLE 2:** Quantification of CC and non-CC neuron clusters in L5 in response to complex sounds.

	A1		AAF		A2		All subfields	
	**non-CC**	**CC**	**non-CC**	**CC**	**non-CC**	**CC**	**non-CC**	**CC**
No. of clusters	1.6 ± 0.4 (7)	4.7 ± 0.9 (20)	1 ± 1 (3)	5.8 ± 1.7 (6)	2.3 ± 0.9 (6)	4.8 ± 1.2 (17)	1.8 ± 0.4 (16)	4.9 ± 0.7 (43)
*p*-value	0.0618		0.1087		0.2323		0.0062	
No. of neurons per cluster	9.7 ± 2 (11)	6.5 ± 0.7 (94)	8.6 ± 2.3 (3)	7.9 ± 1.1 (35)	13.4 ± 3.1 (14)	7.3 ± 1 (82)	11.4 ± 1.8 (28)	7 ± 0.5 (211)
*p*-value	0.0286		0.8332		0.0053		3.2e-04	
Fraction of clustered cells	0.53 ± 0.14 (7)	0.41 ± 0.07 (20)	0.18 ± 0.18 (3)	0.42 ± 0.1 (6)	0.4 ± 0.16 (6)	0.48 ± 0.07 (17)	0.42 ± 0.09 (16)	0.44 ± 0.05 (43)
*p*-value	0.4182		0.2541		0.6274		0.8266	
Phys. distance (μm)	222 ± 6 (695)	348 ± 3 (3,587)	201 ± 14 (116)	301 ± 4 (1,651)	334 ± 5 (2,024)	273 ± 2 (5,151)	301 ± 4 (2,835)	303 ± 2 (10,389)
*p*-value	5.6e-62		2.5e-11		3.3e-25		0.0056	
Correlation	0.13 ± 0.01 (11)	0.28 ± 0.02 (94)	0.16 ± 0.04 (3)	0.24 ± 0.02 (35)	0.17 ± 0.02 (14)	0.32 ± 0.02 (82)	0.15 ± 0.01 (28)	0.29 ± 0.01 (211)
*p*-value	0.0014		0.3247		0.0044		2e-05	
Reliability	0.15 ± 0.01 (11)	0.31 ± 0.02 (94)	0.18 ± 0.04 (3)	0.27 ± 0.03 (35)	0.19 ± 0.02 (14)	0.36 ± 0.03 (82)	0.18 ± 0.01 (28)	0.33 ± 0.01 (211)
*p*-value	0.0137		0.3935		0.0056		1.3e-04	

Data obtained from 6 awake animals. Values are shown as mean ± S.E.M. n-number is depicted in parentheses.

Overall, our results point toward CC neurons forming strong, reliable activity clusters, which span large distances across A1 and AAF, yet smaller or similar distances in A2 when compared to non-CC neurons.

## 4. Discussion

Tonotopy is a hallmark of the auditory system and is preserved from the inner ear through the brainstem up to the AC (Kandler et al., 2009; Kanold et al., 2014). However, up to date very little information about the contribution of different neuron types to tonotopy within the AC has been available. In the present study we used two-photon activity imaging to obtain data that speak for an imprecise tonotopy of L5 CC neurons compared to non-CC neurons in core AC, together with the observation of widespread CC neuron activity clusters which display strong correlations. Furthermore, within A2 CC neurons appeared to be the more topographically ordered neuron type, suggesting subfield differences for the physiological roles of CC neurons.

Regarding frequency tuning of single L5 AC neurons, we found a higher bandwidth for CC neurons compared to non-CC neurons in awake mice. Data obtained by Williamson and Polley (2019) showed a tendency of higher bandwidths in CC neurons compared to the overall L5 population in awake animals. Though this difference was not statistically significant, we still regard this finding to be in line with our data. In general, the number of tuned neurons was lower in anesthetized than in awake animals, and bandwidth was much higher, which can be explained by a very high degree of synchronization of L5 neurons under anesthesia (Bharioke et al., 2022), decreasing specificity of neuronal responses. We thus limited our further analysis mostly to data obtained from awake animals. As already observed by Tischbirek et al. (2019), the amount of well-tuned neurons in AC L5 is quite high, i.e., comparable to layer 2/3. Nevertheless, we observed the proportion to be slightly lower for CC compared to non-CC neurons, fitting with the result of broader tuning.

Anatomical studies from Saldaña et al. (1996) and Bajo and Moore (2005) reported CC projections being topographically organized in rats and gerbils, respectively. This organization is consistent across several species (Stebbings et al., 2014). Nevertheless, those studies only demonstrated that restricted regions of the AC project to limited regions of the IC, e.g., low-frequency region of AC connected with low-frequency region of IC. Lim and Anderson (2007) and Straka et al. (2015) reported CC projections in guinea pigs to be tonotopic using multi-unit recordings to determine BFs in the AC and IC, which leads to a relatively low-resolution description of the regional BF. Thus, so far, the precision of a tonotopic organization of CC neurons remained uninvestigated. Our study provides new insight into this topic. While widefield imaging demonstrated a present tonotopy of CC neurons, single-cell imaging in core AC regions revealed a less precise topography of frequency representation of CC neurons compared to L5 neurons projecting to other–most likely cortical–targets. In contrast, such a difference was not found in A2 when limiting the analysis to well-tuned neurons, which contribute most to AC tonotopy (Gaucher et al., 2020). Furthermore, including all PT-responsive neurons resulted in lower heterogeneity for CC neurons in A2. These findings are in line with CC neuron pairs within activity clusters in response to PTs or complex sounds being on average further apart in A1 and AAF, which is most likely a reflection of less precise tonotopic gradients for CC neurons as well. This approach is, in contrast to local tuning heterogeneity, based on highly activity-correlated neurons within the complete observed population, providing arguably a more physiologically relevant representation of population activity topography. For A2, neuron distance within PT-evoked activity clusters did not differ significantly between the neuron types, with a slight tendency of lower values for CC neurons, which fits to equal or lower local tuning heterogeneity, depending on the subgroup of neurons analyzed. Experiments using PT stimulations also revealed that CC neurons formed large clusters containing many neurons and had the tendency of displaying more reliable responses than non-CC neurons. This is in line with a higher tuning bandwidth of CC neurons in awake animals discussed above as well as with the idea of the broadcast function of corticofugal neurons concluded by e.g., Williamson and Polley (2019), who observed low sound selectivity of CC neurons. We conclude that CC neurons in core AC feature a weaker tonotopic organization than cortical-projecting pyramidal neurons of AC L5. For A2, topographic order of CC neurons appears to be more complex and should be investigated further in future studies.

It should be noted that a small fraction of auditory corticofugal L5 neurons project to subcollicular targets but not the IC, which thus probably contaminated the imaged cortical-projecting population in our experiments slightly. However, from the work by Doucet et al. (2003), it can be estimated that only about 10% of ipsilateral-projecting corticofugal neurons target the cochlear nucleus or superior olivary complex. While this does not provide a complete estimation of the number of subcollicular-projecting neurons, it can still be concluded that the non-CC population imaged consisted of to a vast majority of cortical-projecting (intratelencephalic) neurons. This is also confirmed by us observing about half of L5 pyramidal neurons being CC neurons, in line with the reported fraction of intrinsic-bursting neurons (Sun et al., 2013), demonstrating that most of them project to the IC.

The idea of an imprecise tonotopy of CC neurons would be in agreement with several observations made in other studies. An imaging study in the IC reported that CC boutons in the dorsal cortex of the IC (DCIC) in some animals studied are tonotopically ordered, forming a gradient similar to the DCIC tonotopy (Barnstedt et al., 2015). However, they also observed a complete lack of such an organization in other mice. Additionally, the existence of responsive boutons was sparse, and responses were unreliable, suggesting that DCIC neurons receive receptive field properties from other brainstem nuclei, while the cortical input provides rather modulatory effects. However, the tonotopic organization of the DCIC is debated, and thus the topographic arrangement of CC neuron boutons in this region is difficult to directly relate to our data. A recent study showed that, from all recorded central IC neurons which receive input from the AC, only 33% are frequency-matched (Qi et al., 2020), which is a hint supporting the statement that linear characteristics are of minor importance at this level of processing. It seems reasonable that basic modulations like BF shifts are carried out by neurons with clear shaped FRAs, and the number of BF-matched L5 CC connections might actually not be sufficient to carry out this task. Consequently, those modulations, where a precise tuning is necessary, would rather be carried out by L6 CC neurons, as their synaptic input was shown to be very similar to L6 corticothalamic neurons (Slater et al., 2018), which are known to express a narrower frequency tuning, and a higher stimulus sparsity compared to L5 CC neurons (Williamson and Polley, 2019). On the other hand, Yudintsev et al. (2021) found L5 CC neurons to be concentrated in AC subfields, while L6 CC neurons were more widely distributed across the temporal cortex. They thus concluded that L6 CC neurons integrate complex, multisensory information, while L5 CC neurons perform frequency-specific actions. Furthermore, cluster analysis of our data obtained from complex sound stimulation actually shows a large number of small CC neuron clusters, which might be interpreted as a high capability of categorizing complex sounds, in contrast to a low capability to categorize PTs. Williamson and Polley (2019) observed low sound selectivity of CC neurons, but the sounds they used were not natural, yet artificially created with varying carrier and modulation frequencies, BWs, and sound pressure levels. While certainly more complex than PTs, the direct comparison to our results using natural animal vocalizations would nevertheless not be warranted. Furthermore, comparing the size of CC and non-CC clusters might in this specific instance be difficult due to differences in correlation. When observing activity of L5 neurons on a large scale over several cortical areas, it was found that corticofugal neurons are important for behavioral reactions, yet less important than other L5 neurons for analyzing sensory stimuli (Musall et al., 2023). Thus, observing topographical CC activity patterns in a behavioral (possibly learning-related) context might be the only way to definitively describe their role in providing frequency-specific feedback to the IC. Ford et al. (2022) imaged activity in dendrites of CC neurons during behavior and concluded that they contribute to learning by transmitting non-auditory signals. However, they did not report on topographical aspects. Furthermore, imaging activity patterns of L6 CC neurons would be of high interest to analyze their tonotopic order. This was however, not possible in the present study, as GCaMP expression was not observed outside of L5 after injection of retrogradely labeling AAV into the IC. This tropism has been observed before after injection into the thalamus, while labeling of L6 neurons could be achieved using rabies virus-based approaches (Gu et al., 2023).

Interestingly, subfield-specific differences were also found when comparing CC and non-CC neurons activity clusters. In A2, physical distance within PT activity clusters were similar between the two neuron types, and the physical distance within activity clusters was actually lower for CC neurons when analyzing data obtained using complex sounds. While these findings suggest substantial differences in the computation of CC neurons in A2 compared to A1 and AAF, we can only speculate about the functional implications at this point. A2 networks and/or neurons have recently been shown to integrate two-tone and multifrequency sounds, preferentially encoding harmonic sounds (Kline et al., 2021, 2023), and to display a high degree of plasticity of OFF-responses in relation to frequency modulations and pup vocalizations (Chong et al., 2020) as well as more categorization of sounds than lower AC fields (Yin et al., 2020). While A1 is thought to code mainly for the spectral component of sounds (Solyga and Barkat, 2021), A2 is associated with encoding complex sounds (Carruthers et al., 2015). It thus makes sense that for core AC large spatial distances between CC neurons within activity clusters evoked by complex sounds mirrors high local tuning heterogeneity of well-tuned neurons, while for A2 the topography of well-tuned neurons has little effect on the topography of complex sound-evoked activity clusters, because here other sound aspects than frequency determine CC neuron response properties. High topographic order of A2 CC neurons is also reflected in local tuning heterogeneity being low when including all PT-responsive neurons, yet whether that is in direct relation to spatially restricted complex sound-evoked topography is unclear at this point. Furthermore, task-relevant sounds modulate activity level stronger in A2 than A1 (Atiani et al., 2014), and projections from the higher-order auditory thalamus to the AC, which are involved in associative memory, are more pronounced in A2 than A1 (Pardi et al., 2020). Given these complex, high-order computations reported for A2 it stands to reason that A2 L5 CC neurons might provide more specific tasks than those within A1 and AAF, which our data hint at. Further studies will be needed to provide detailed insight into this matter.

## 5. Conclusion

In conclusion, our study demonstrates differences in population activity and frequency response-related topographic organization between CC and non-CC neurons in L5 AC. Weaker tonotopy concluded for CC neurons in core subfields is in line with low sound selectivity, yet for A2 topographic order appears to be more complex. Our findings indicate that AC tonotopy is neuron type- and subfield-dependent, adding further to the complexity of its topographical organization.

## Data availability statement

The raw data supporting the conclusions of this article will be made available by the authors, without undue reservation.

## Ethics statement

The animal study was reviewed and approved by the Landesuntersuchungsamt Rheinland-Pfalz.

## Author contributions

TS and KA acquired data. JH wrote the manuscript. All authors analysed data and provided custom-written code.

## Footnotes

1
http://n2t.net/addgene:104488

2
http://n2t.net/addgene:59462

3
http://www.avisoft.com/animal-sounds/

4
https://micro-manager.org

5
https://github.com/MouseLand/suite2p
