# Supplementary material for: Distinct topographic organization and network activity patterns of corticocollicular neurons within layer 5 auditory cortex
Source: Front Neural Circuits. 2023 Jul 13;17:1210057. doi: 10.3389/fncir.2023.1210057 (PMC10372447; doi:10.3389/fncir.2023.1210057)
Supplement: Supplementary file 1 [file Data_Sheet_1.pdf]

Supplementary Material

Distinct topographic organization and network activity patterns of corticocollicular neurons within layer 5 auditory cortex

Tatjana T.X. Schmitt, Kira M.A. Andrea, Simon L. Wadle, and Jan J. Hirtz*

*** Correspondence:** Jun.-Prof. Dr. Jan J. Hirtz: hirtz@bio.uni-kl.de

Supplementary Figures

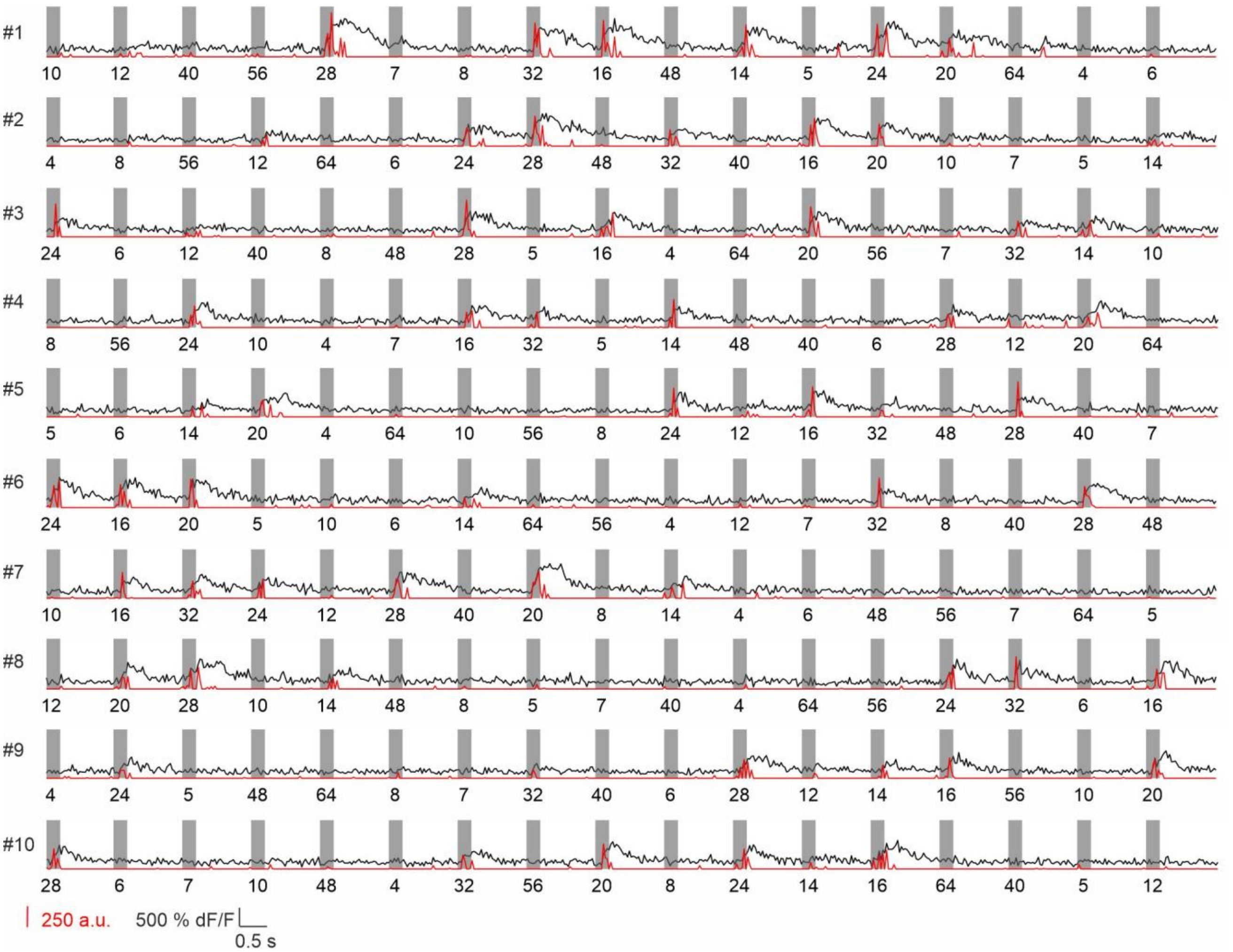

Supplementary Figure 1. Raw fluorescence trace and deconvolution. Uncut extracted fluorescent activity (dF/F, black trace) from a CC neuron recorded under awake conditions and corresponding deconvolution (red trace) during PT presentation at 70 dB SPL. Numbers indicate frequency in kHz. Timings and length (250 ms) of presented PTs are illustrated by grey bars with the corresponding

frequency depicted above. Each row denotes one repetition, containing each of the 17 PTs once. GCaMP7f expression was achieved by injection of AAV2-retro-hSyn-GCaMP7f into the IC.

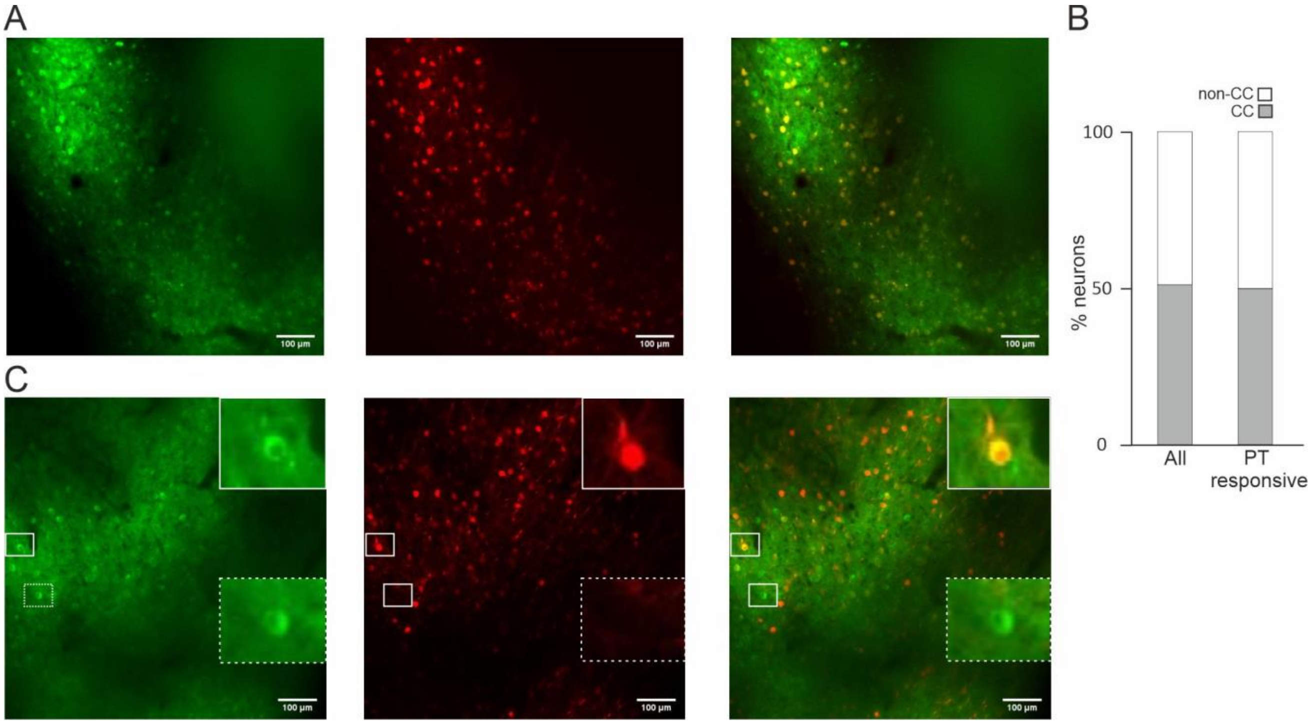

Supplementary Figure 2. Identifying non-CC and CC neurons using the double labeling approach. **(A)** FOV within the AC of an animal expressing GCaMP7f in all pyramidal neurons, as seen in the green channel (left), and tdTomato in the red channel (middle). On the right, both channels are overlayed. **(B)** Overall proportion of non-CC and CC neurons observed with the approach displayed in A, for all active and PT-responsive neurons, 13 FOVs analyzed in 3 animals. **(C)** As A, but also showing magnifications of a neuron determined as CC (solid white square) and non-CC (dotted white square).

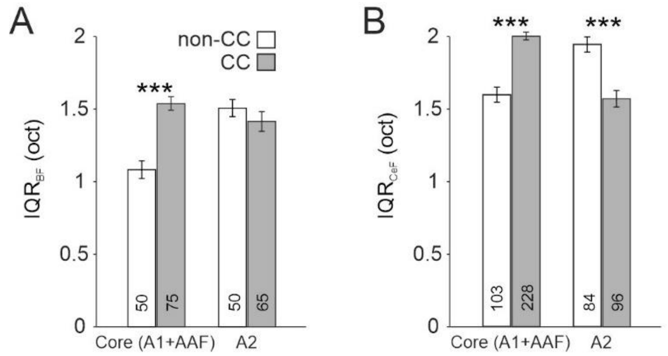

Supplementary Figure 3. Local tuning heterogeneity of L5 non-CC and CC neurons, extended. **(A)** Quantification of IQR_{BF} within core subfields and A2. Before calculation, 50-80% of CC neurons were randomly excluded. **(B)** As A, but for CeF.
